# Relative efficacy of topical non-steroidal anti-inflammatory drugs and topical capsaicin in osteoarthritis: protocol for an individual patient data meta-analysis

**DOI:** 10.1186/s13643-016-0348-8

**Published:** 2016-09-29

**Authors:** Monica S. M. Persson, Yu Fu, Archan Bhattacharya, Siew-Li Goh, Marienke van Middelkoop, Sita M. A. Bierma-Zeinstra, David Walsh, Michael Doherty, Weiya Zhang, OA Trial Bank Consortium

**Affiliations:** 1 Academic Rheumatology, Clinical Sciences Building, University of Nottingham, City Hospital, Nottingham, UK; 2 Arthritis Research UK Pain Centre, Nottingham, UK; 3 School of Healthcare, University of Leeds, Leeds, UK; 4 Arthritis UK Centre for Sports, Exercise and Osteoarthritis, Nottingham, UK; 5 Sports Medicine Unit, University of Malaya, Kuala Lumpur, Malaysia; 6 Department of General Practice, Erasmus MC Medical University Center Rotterdam, Rotterdam, The Netherlands

**Keywords:** Osteoarthritis, Topical, NSAIDs, Capsaicin, Individual patient data meta-analysis

## Abstract

**Background:**

Pain is the most troubling issue to patients with osteoarthritis (OA), yet current pharmacological treatments offer only small-to-moderate pain reduction. Current guidelines therefore emphasise the need to identify predictors of treatment response. In line with these recommendations, an individual patient data (IPD) meta-analysis will be conducted. The study aims to investigate the relative treatment effects of topical non-steroidal anti-inflammatory drugs (NSAIDs) and topical capsaicin in OA and to identify patient-level predictors of treatment response.

**Methods:**

IPD will be collected from randomised controlled trials (RCTs) of topical NSAIDs and capsaicin in OA. Multilevel regression modelling will be conducted to determine predictors for the specific and the overall treatment effect.

**Discussion:**

Through the identification of treatment responders, this IPD meta-analysis may improve the current understanding of the pain mechanisms in OA and guide clinical decision-making. Identifying and prescribing the treatment most likely to be beneficial for an individual with OA will improve the efficiency of patient management.

**Systematic review registration:**

CRD42016035254

**Electronic supplementary material:**

The online version of this article (doi:10.1186/s13643-016-0348-8) contains supplementary material, which is available to authorized users.

## Background

Osteoarthritis (OA) is the most common joint disorder [1] and frequently affects the knee, hand, foot and hip joints [2]. Risk factors for the development of OA include ageing, female gender, obesity, genetic factors, occupation, increased bone density and local factors such as previous joint injury and joint shape or malalignment [1–3]. OA is estimated to affect approximately 8.75 million people [2] and is the 11th largest contributor to years lived with disability in the UK [4]. Pain is often the most troubling issue to individuals with OA [5, 6] and is the most common reason to present to the general practitioner (GP) [1].

The nature of the pain in OA is variable, and this may serve to further our understanding of the underlying pain mechanisms. Hawker et al. found that OA pain, particularly in the early stages, is strongly mechanical in nature and is brought on in a predictable fashion by certain activities [6]. In more advanced disease, the pain is often described as a continuous, dull ache interspersed with shorter episodes of unpredictable, sharp pain [6]. A subset of patients with OA also describes burning, shooting or tingling pain that may be neuropathic in nature [6–8]. Features of central sensitization, such as allodynia [8], increased temporal summation, and impaired conditioned pain modulation [9], have also been identified in patients with OA. Furthermore, person-specific characteristics such as catastrophizing, anxiety or depression can also modify pain experience [10].

Within the recommendations endorsed by the National Institute for Health and Care Excellence (NICE), the European League Against Rheumatism (EULAR), Osteoarthritis Research Society International (OARSI), and the American College of Rheumatology (ACR), over 10 different pharmacological management options are recommended for OA pain [1, 11–14]. However, the difference between treatment and placebo for commonly recommended pharmaceutical treatments was found to be 0.39 (95 % CI 0.31–0.47) [15], which is only equivalent to about 10 % pain reduction [16].

It is possible that the modest effect size of OA treatments arises from a mismatch between a patient’s predominant pain mechanism and the treatment most effective for that mechanism. For this reason, treatment guidelines have emphasised the need to identify predictors of treatment response in order to tailor treatment to the individual patient [11, 12, 17]. Identifying treatment responders according to the presence of certain clinical or investigational features would have two benefits. Firstly, it would allow clinicians to select the treatment that is most likely to benefit an individual patient. This is in keeping with the principle in precision medicine whereby understanding patient variability is considered to increase the efficiency of patient management [18]. Secondly, it may expand our understanding of the mechanisms that underlie the pain experience in OA.

Two drugs recommended by the majority of guidelines for hand and knee OA are topical non-steroidal anti-inflammatory drugs (NSAIDs) and topical capsaicin [1, 11–14]. With regard to pain reduction, meta-analyses of topical NSAIDs have shown a small-to-moderate effect size compared to placebo [19–22]. NSAIDs are thought to act by inhibiting the cyclooxygenase enzymes responsible for the production of potent hyperalgesic mediators [23, 24]. Topical capsaicin also appears to be superior to placebo for pain relief in OA [25–27] and is believed to act by causing defunctionalisation of nociceptive fibres [28, 29]. As these topical treatments act by different mechanisms, the comparison between the two treatments may advance our understanding of the underlying pain processes in OA. In addition, understanding the characteristics that make a patient more likely to respond to either of these treatments may also serve to guide our choice of treatment for OA patients in the future. Although several meta-analyses have shown that topical NSAIDs and capsaicin are efficacious in OA, little is known about who responds better to the treatments and what the predictors of response to these treatments are in OA.

The primary aim for this study is to investigate the relative treatment effects of topical NSAIDs and topical capsaicin in OA and to identify patient-level predictors of treatment response.

## Methods/design

We will conduct an individual patient data (IPD) meta-analysis of randomised controlled trials (RCTs) to investigate the efficacy and predictors of topical NSAIDs or topical capsaicin in individuals with OA. This is an alternative method that increases the power of subgroup analysis compared to a single RCT. In addition, we will conduct a network meta-analysis to examine the relative effect between topical NSAIDs and topical capsaicin.

### Study selection

#### Type of studies

Published and unpublished RCTs that evaluate the efficacy of either topical NSAIDs or topical capsaicin in participants with OA will be included. There will be no language or geographical restrictions applied to the studies.

#### Participants

Men and women with physician diagnosed OA, as defined by the criteria endorsed by the ACR [30, 31]. Participants with chronic joint pain, not due to rheumatoid arthritis or other forms of arthritis, will also be included. Studies investigating only OA patients and studies with a subgroup of OA patients will be included, as long as IPD data can be collected separately for the OA participants. Participants with back pain and other arthritic pain, such as rheumatoid arthritis pain, will be excluded.

#### Types of interventions

Only topical NSAIDs or topical capsaicin will be studied. These can include any formulation, dosage or drug within the group. Salicylates, although pharmacologically related to NSAIDs, will be excluded as their principal action when applied topically is different from that of topical NSAIDs [32]. Interventions involving ultrasound or magnetophoresis of the topical treatment will also be excluded. Controls will include placebo, active comparators, continuing usual treatment, no treatment and head-to-head comparison between topical NSAIDs and topical capsaicin. Both open label and blinded studies will be included.

#### Types of baseline assessments

As a minimum, studies will need to have assessed the level of pain and important patient characteristics, including age and gender, at baseline.

#### Types of outcomes

For inclusion, trials must have an adequate assessment of pain. As recommended by the OMERACT-OARSI Initiative [33], measures of functional impairment and patient’s global assessment will also be included in analysis when available.

Duration of follow-up will be a minimum of 1 week, and the primary outcome will be measured at 4 weeks or closest to 4 weeks.

### Identification of eligible studies

A systematic database search of Medline (OVID interface, 1946 onwards), Embase (OVID interface, 1974 onwards), AMED (OVID interface, 1985 onwards), CINAHL (EBSCOhost interface, all years), Web of Science (Core Collection, 1900 onwards) and the Cochrane library (Wiley interface, current issue) will be conducted to identify all relevant RCTs of topical NSAIDs or topical capsaicin in OA up until 16 November 2015. The search strategies are based on those from the Cochrane reviews of topical NSAIDs [24] and topical capsaicin [29, 34], with the inclusion of other terms found through searching the internet. The search strategies used can be found in Additional file 1. Further studies will also be identified by contacting pharmaceutical suppliers of topical NSAIDs and topical capsaicin, identified via the British National Formulary, the electronic Medicines Compendium and Clinicaltrials.gov, to ask for any unpublished trial data. Reference lists of included trials and reviews in the area will also be hand-searched for any trials not found from searching the databases. Review authors and collaborating authors will be asked if they are aware of any eligible studies not yet included.

References will be exported to Endnote, where duplicates will be removed and eligibility for inclusion can be assessed. One of the investigators (MP) will assess if studies meet the inclusion criteria by screening the titles, the abstracts and finally the full texts of articles found through the systematic search. The list of included and excluded studies will then be independently reviewed by another investigator (YF), who will assess all full texts. Any disagreements regarding the inclusion of studies will be discussed between the two review authors. If no consensus can be reached, a third investigator will be consulted.

### Data collection

The primary or corresponding author for all eligible trials will be contacted and asked to collaborate on the project. If they cannot be contacted, all co-authors listed will be emailed. If none of the authors can be reached, the institution where the research was carried out will be contacted. Authors or institutions interested in collaborating will be asked for access to the IPD from the original study. The data will be accepted in any format and will be transferred into Microsoft Access for storage. The data will be contributed to the OA Trial Bank by the authors, as described by van Middelkoop et al. [35].

### Data extraction

Data extracted from the published reports will include the distribution of participant demographics and disease characteristics, study information and design, details of the intervention and outcome measures. These will be entered into a data extraction form by MP and will be independently verified by another investigator.

Data contributed by the original study authors will include patient characteristics such as age, gender and body mass index (BMI). If available, other characteristics will be collected including duration of complaints, radiographic change, level of inflammation (e.g. clinical or ultrasound effusion, CRP), type of pain (e.g. neuropathic-like pain through PainDetect), central sensitization (e.g. pain elsewhere through a questionnaire or pain threshold through quantitative sensory testing) and psychological assessments (e.g. depression, anxiety, sleep disturbance, catastrophizing). Study-level characteristics, such as setting (community or hospital), allocation concealment, blinding, sample size, risk of bias (low vs. high), intervention(s) used, control(s) used, doses and formulations and duration of follow-up will also be required. Finally, authors will be asked to contribute the outcome measures of pain, function and patient’s global assessment at baseline and at all subsequent assessments.

All participants that have been randomised will be included in the pooled database and will be included in intention-to-treat (ITT) analyses. Secondary per-protocol analyses will also be carried out.

### Quality assessment

The quality of included studies will be assessed using a modified version of the risk of bias tool recommended by the Cochrane Collaboration [36] (Table 1 and Additional file 2). It is composed of nine questions that assess each of the domains set out in the risk of bias tool. In contrast with the narrative nature of the Cochrane tool, the modified version uses questions that are scored as yes, no or unclear. Each question evaluates the risk of bias in an objective manner, so as to minimise the inter-rater disagreement that is associated with the more subjective domains in the risk of bias tool [37]. As the reviewer carrying out the quality assessment will vary by the language of the study, these modifications were aimed at reducing inter-rater variability in the quality scoring.

**Table 1:**
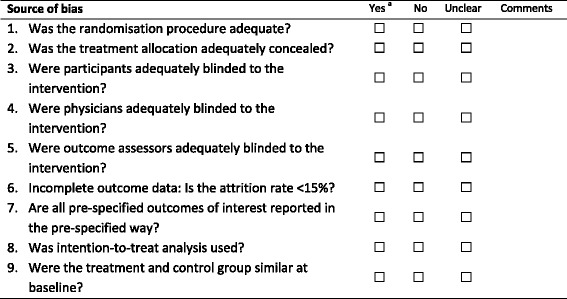
Modified risk of bias assessment

Modified from Cochrane’s risk of bias tool [36]

^a^See Additional file 2 for criteria for a judgement of “yes”

The first five questions of the modified tool assess selection bias, performance bias and detection bias. Each of these questions will be scored using the criteria outlined by Cochrane [36]. The sixth question is a measure of attrition bias, and similarly to the PEDro and van Tulder quality scales [38], a cut-off for acceptable drop-out rates was used to determine the risk. After discussion between review authors, a consensus of 15 % cut-off for attrition bias was agreed upon [39]. The seventh question addresses selective reporting by comparing the outcomes pre-specified in the methods with those presented in the results. Within the domain “Other bias”, two questions cover the use of intention-to-treat analysis and whether the treatment and control groups were similar at baseline. Although not pre-specified in Cochrane’s risk of bias [36], these two sources of bias are assessed in a variety of other RCT quality scales [38].

Similar to the protocol of van Middelkoop et al. [35], each study will be categorised as “low risk of bias” if it meets at least five of the criteria items in the list. Studies will then be categorised as “low risk of bias”, “high risk of bias” or “unclear”. With the exception of non-English publications, the quality assessment of each study will be undertaken independently by two authors. If the data extracted is from a study in a language not spoken by either or both authors, the risk of bias assessment will be done only by the author or a colleague that speaks the language. The risk of bias score will be used to assess study-level predictors of response.

In addition, the quality of the IPD data will be assessed. The quality indicators include number of trials eligible per treatment, number of trials with the IPD data obtained and percentage of the data obtained per trial.

In order to determine how representative the IPD analysis is, a comparison will be made of the patient characteristics between all eligible trials and those where IPD was obtained. Characteristics of studies that are not included, due to missing raw data, unwillingness to collaborate or inability to reach study authors, will be presented and compared to those of the included studies. Furthermore, sensitivity analyses will be conducted to compare the summary effects of the included studies with the summary effects extracted from the published reports of the studies that were not included. Finally, assessments of funnel plot asymmetry will be conducted to assess for publication bias.

### Data analysis

Once data have been received, the databases will be cleaned and merged. Means and standard deviations will be used to describe normally distributed continuous data, medians and interquartile ranges will be used to describe continuous data that are not normally distributed, and frequencies and percentages will be used for categorical data. Data will be described using 95 % confidence intervals. *P* values <0.05 will be considered significant. Any missing data will be presumed to be missing at random; therefore, a multiple imputation method will be used within each trial before pooling the data. *I*^2^ will be calculated as a measure of the heterogeneity of the included trials. Data analysis will be conducted using the statistical program Stata SE 14 (StataCorp, College Station, Texas).

The primary outcome will be pain at 4 weeks of treatment duration or closest to 4 weeks. Secondary outcomes will include pain at other durations of follow-up, area under the curve (AUC) for pain scores at the different time points, function and global assessment measures.

A conventional meta-analysis will be undertaken using study-level variables such as mean pain score, mean age, mean BMI and sample size. This analysis will help to determine the treatment effect and its variation between studies. A network meta-analysis will be conducted to determine the relative efficacy between topical NSAIDs and capsaicin. Finally, an IPD meta-analysis will be conducted, using both study-level and IPD-level variables, to determine the treatment effect and its potential predictors. Both a one-stage (primary) and two-stage (secondary) approach will be used, and a sensitivity analysis will be undertaken to compare the findings.

#### Conventional meta-analysis

##### Overall analysis

An aggregate data meta-analysis, using a random-effects model, will be performed to estimate the treatment effect of topical NSAIDs and topical capsaicin over placebo. This will be used for the sensitivity analyses comparing the treatment effect in the studies included in the IPD analysis with all eligible studies. The relative efficacy between the two drugs will be examined directly by comparing topical NSAIDs and topical capsaicin within any available head-to-head comparison trials and indirectly by conducting a network meta-analysis using a common comparator.

##### Subgroup analysis

When sufficient data are available, subgroup analysis will be performed for the primary outcome—pain at 4 weeks according to pain elsewhere (yes/no), type of pain (dull/neuropathic), pain severity (low/high intensity), disease severity (marked/modest disease radiographically), level of inflammation (low/high) and duration of pain.

##### Network meta-analysis

A number of trials have been undertaken for topical NSAIDs and topical capsaicin in OA. However, there are no head-to-head comparisons between these two commonly used topical analgesics. Due to the different mechanisms of actions, we assume that topical capsaicin may be better than NSAIDs in neuropathic-like pain, such as post-herpetic neuralgia, diabetic neuropathy and end-stage stage OA with more neuropathic damage. This begs a comparison between these two agents in different pain models. A network meta-analysis has therefore been proposed [40].

We will use topical placebo as a common comparator (or “node”) to network topical NSAIDs and topical capsaicin in each condition to calculate the relative difference between the two active treatments. To maximise the information, we will also include trials comparing topical NSAIDs or capsaicin (if any exist) with oral NSAIDs or other analgesics. In this scenario, oral NSAIDs or other analgesics will be the “node” to link topical NSAIDs and topical capsaicin. If needed, more than one “node” will be used to link the two treatments, and a more complicated network may be developed.

Once the network is developed, the relative efficacy between topical NSAIDs and topical capsaicin will be calculated. Statistical pooling will be undertaken taking into consideration the variations between studies and comparisons. In order to increase the precision of the estimate, the Bayesian statistical approach will be applied [41].

#### IPD meta-analysis

##### Two-stage modelling

A regression model, adjusted for baseline pain severity using an analysis of covariance (ANCOVA) method, will be developed for each trial and the interaction/predictor terms from these models will be pooled between trials. Depending on the definition of the treatment effect (dependent variable), the model will be built with or without interaction terms in order to identify the predictors. For the specific treatment effect (i.e. the difference between treatment and placebo), an interaction term between treatment and potential predictor will be used to define the predictors of treatment response. The partial regression coefficient of this interaction will be selected and pooled with the same interaction terms obtained from the other trials. For the overall treatment response (i.e. improvement from baseline), the prediction model is developed within each arm; therefore, there is no need to use an interaction term to identify a predictor. In this case, the partial regression coefficient of the predictor will be used and pooled between trials. The interaction/predictor terms will be pooled using a random-effects meta-analytic technique, based on the inverse-variance method.

##### One-stage modelling

Treatment responders will be determined using a one-step random-effects IPD meta-analysis approach, i.e. taking into account both study-level and individual patient-level covariates in the regression model. All variables listed under the “Data extraction” section will be considered potential predictors. Individual patient-level covariates will be centred to the mean of the covariate in each trial. In order to quantify the presence of ecological bias, the study-specific mean of the covariate will also be used. The overall treatment effect (i.e. change from baseline in participants in the treatment group) and specific treatment effect (i.e. difference between treatment and placebo) will be calculated [42]. Two multilevel regression models will be developed to consider patient-level and study-level (i.e. cluster) effects, one to examine predictors of the specific treatment effect and the other to examine the predictors of the overall treatment effect. Models will be built with one potential predictor and interaction term (random-effects), and will be adjusted for trial (random intercept), baseline pain score and other covariates (fixed effects).

The first model will include participants from both intervention groups where the specific treatment effect (i.e. change from baseline in both groups) will be the dependent variable and treatment (active or placebo) and patient characteristics will be independent variables. The interaction between the treatment and predictor will be used to identify the effect modifiers.

The second model will only include participants in the treatment group, where the overall treatment effect will be the dependent variable (i.e. change from baseline in treatment group) and patient characteristics will be independent variables. This model is based on the assumption that any treatment effect includes both specific and non-specific contextual effects (i.e. placebo effect) [42], and in clinical practice, we only give treatment not placebo. The aim of this model is to identify the treatment responders and factors related to the response.

##### Exploratory analyses

Identifying treatment responders in a clinical setting, where placebo is not used, requires careful examination of the differences between baseline pain scores and endpoint pain scores. A receiver operating characteristic (ROC) curve will be used to find the cut-off point that gives the best separation between baseline and endpoint scores, i.e. the most sensitive and specific threshold. Alternatively, a threshold of 20 % pain reduction from baseline may be used [43]. Once a threshold for response has been established, a binomial model will be developed as appropriate. The model will be built as described in “One-stage modelling” but will use logistic regression and responder/non-responder as the dependent variable.

##### Potential predictors

All baseline variables described in the “Data extraction” section will be considered potential predictors of treatment response. As a minimum, we will utilise age, gender, BMI, baseline pain, pain elsewhere, evidence of inflammation (clinical or imaging) and radiographic findings as patient-level variables. For study-level variables, we will include joint treated, sample size, setting (community/hospital) and allocation concealment as a minimum.

## Discussion

An IPD meta-analysis forms a part of precision medicine. It helps identify potential predictors of treatment response at a patient level, which is not possible with conventional meta-analyses. As the IPD meta-analysis combines multiple trials with the same treatment and control, it increases the power of the study, thereby permitting subgroup analysis. Individual trials, on the other hand, are often not powered for these types of analyses. Conducting IPD analyses has therefore become very popular, and a proposal by the International Committee of Medical Journal Editors (ICMJE) aims to incorporate it in the publication criteria for trials. As such, trials may only be published if the de-identified IPD are made available within 6 months of publication [44].

There are, however, some caveats associated with analysis. Firstly, not all predictors of response have been collected in the existing trials. This is the case, even for trials with the same treatment and control groups, as every trial has its own hypothesis and primary outcome measures. It is very difficult to acquire data for a specific question from all studies, especially data related to subgroup indicators. This limits the IPD analysis to predictors that are available in the eligible studies. Secondly, it may not be possible to contact original trial authors, authors may not be willing to collaborate, or they may not have access to the raw data required [45, 46]. These difficulties, if not overcome, may introduce bias to the meta-analysis if the studies not included are systematically different from those included [45–47]. Several methods for overcoming these difficulties will be used, including providing de-identified data [46], facilitating the process of data collection for original authors by accepting the data in any format or manner [45, 47] and pooling resources in the form of collaborative groups [46]. We hope that in the near future, an organisation or databank, like the Cochrane Library, will be developed for IPD as this will better the field of evidence-based medicine.

## Additional files

Additional file 1: Search strategy examples. Example of search strategy used in Medline. (DOCX 15 kb) Additional file 2: Modified risk of bias tool. Assessment criteria for scoring the modified risk of bias tool. (DOCX 22 kb)

## Abbreviations

ACR: American College of Rheumatology
ANCOVA: Analysis of covariance
AUC: Area under the curve
BMI: Body mass index
CRP: C-reactive protein
EULAR: European League Against Rheumatism
GP: General practitioner
IPD: Individual patient data
ITT: Intention-to-treat
NICE: National Institute for Health and Care Excellence
NSAID: Non-steroidal anti-inflammatory drug
OA: Osteoarthritis
OARSI: Osteoarthritis Research Society International
RCT: Randomised controlled trial
ROC: Receiver operating characteristic
