# Supplementary material for: Relative efficacy of topical non-steroidal anti-inflammatory drugs and topical capsaicin in osteoarthritis: protocol for an individual patient data meta-analysis
Source: Syst Rev. 2016 Sep 29;5:165. doi: 10.1186/s13643-016-0348-8 (PMC5043618; doi:10.1186/s13643-016-0348-8)
Supplement: Additional file 1: — Search strategy examples. Example of search strategy used in Medline. (DOCX 15 kb) [file 13643_2016_348_MOESM1_ESM.docx]

**Additional file 1. Search strategy examples**

| **OA and NSAID search strategy: Medline (via Ovid)** |
| --- |
| 1. Randomized Controlled Trials as Topic/  2. (randomi?ed controlled trial or double-blind or blind$ or mask$ or clinical trial or trial).af.  3. Administration, Topical/  4. (stick-on or cutaneous or dermal or transcutaneous or percutaneous or skin or massage or embrocation or gel or ointment or aerosol or cream or lotion or mousse or foam or liniment or spray or rub or balm or salve or emulsion or oil or patch or plaster).af.  5. exp Osteoarthritis/dt, th [Drug Therapy, Therapy]  6. (osteoarthr* or OA or arthritis or Degenerative arthritis or degenerative joint disease or arthrosis or osteoarthrosis).af.  7. Anti-Inflammatory Agents, Non-Steroidal/ad, ae, tu [Administration & Dosage, Adverse Effects, Therapeutic Use]  8. (nsaid or nonsteroidal antiinflammatory or non-steroidal anti-inflammatory or bufexamac or bufexine or calmaderm or ekzemase or dicoflenac or solaraze or pennsaid or voltarol or emulgen or voltarene or optha or voltaren or etofenamate or afrolate or algesalona or bayro or deiron or etofen or flexium or flogoprofen or rheuma-gel or rheumon or traumalix or traumon or zenavan or felbinac or dolinac or flexfree or napageln or target or traxam or fentiazac or domureuma or fentiazaco or norvedan or riscalon or fepradinol or dalgen or flexidol or cocresol or rangozona or reuflodol or pinazone or zepelin or flufenamic or dignodolin or rheuma or lindofluid or sastridex or lunoxaprofen or priaxim or flubiprofen or fenomel or ocufen or ocuflur or "trans act lat" or tulip or ibuprofen or cuprofen or "deep relief" or fenbid or ibu-cream or ibugel or ibuleve or ibumousse or ibuspray or "nurofen gel" or proflex or motrin or advil or radian or ralgex or ibutop or indomethacin or indocin or indospray or isonixin or nixyn or ketoprofen or tiloket or oruvail or powergel or solpaflex or ketorolac or acular or trometamol or meclofenamic or naproxen or naprosyn or niflumic or actol or flunir or niflactol topico or niflugel or nifluril or oxyphenbutazone or californit or diflamil or otone or tanderil or piketoprofen or calmatel or triparsean or piroxicam or feldene or pranoprofen or oftalar or pranox or suxibuzone or danilon or flamilon or ufenamate or fenazol).af.  9. 1 or 2  10. 3 or 4  11. 5 or 6  12. 7 or 8  13. 9 and 10 and 11 and 12  14. Limit 13 to randomized controlled trial |
| **Citations found: 141** |
| **OA and capsaicin search strategy: Medline (via Ovid)** |
| 1. Randomized Controlled Trials as Topic/  2. (randomi?ed controlled trial or double-blind or blind$ or mask$ or clinical trial or trial).af.  3. Administration, Topical/  4. (stick-on or cutaneous or dermal or transcutaneous or percutaneous or skin or massage or embrocation or gel or ointment or aerosol or cream or lotion or mousse or foam or liniment or spray or rub or balm or salve or emulsion or oil or patch or plaster).af.  5. exp Osteoarthritis/dt, th [Drug Therapy, Therapy]  6. (osteoarthr* or OA or arthritis or Degenerative arthritis or degenerative joint disease or arthrosis or osteoarthrosis).af.  7. Capsaicin/ad, ae, tu [Administration & Dosage, Adverse Effects, Therapeutic Use]  8. (8-Methyl-N-Vanillyl-6-Nonenamide or 8 methyl n vanillyl 6 nonenamide or axsain or capsaicin or capsaicine or capsicum farmaya or capsidol or capsin or capzasin or gelcen or katrum or ngx 4010 or ngx-4010 or ngx4010 or zacin or zostrix or capsici or capsig or capsina or capsiplast or capzasin-p or dolorac or no pain-hp or priltam or r-gel or chilli or chili).af  9. 1 or 2  10. 3 or 4  11. 5 or 6  12. 7 or 8  13. 9 and 10 and 11 and 12  14. Limit 13 to randomized controlled trial |
| **Citations found: 7** |
