# Supplementary material for: Relative efficacy of topical non-steroidal anti-inflammatory drugs and topical capsaicin in osteoarthritis: protocol for an individual patient data meta-analysis
Source: Syst Rev. 2016 Sep 29;5:165. doi: 10.1186/s13643-016-0348-8 (PMC5043618; doi:10.1186/s13643-016-0348-8)
Supplement: Additional file 2: — Modified risk of bias tool. Assessment criteria for scoring the modified risk of bias tool. (DOCX 22 kb) [file 13643_2016_348_MOESM2_ESM.docx]

**Additional File 2. Modified Risk of Bias tool**

| **Source of bias** | **Criteria for a judgment of “yes”** | **Criteria for a judgment of “no”** |
| --- | --- | --- |
| 1. **Was the randomisation procedure adequate?** | ***YES*** *– A random component in the sequence generation process is used, such as:*   - *Using to a random number table* - *Using a computer random number generator* - *Tossing a coin* - *Shuffling cards or envelopes* - *Throwing dice* - *Drawing lots* - *Minimisation* - *Randomisation generated by outside statistician/central group* - *Random permuted block scheme* | ***NO*** *– a non-random component is used in the allocation process, such as:*   - *Sequence generated using odd or even date of birth* - *Sequence based on a rule based on date of admission or hospital number* - *Allocation decision is made by clinician or participant* - *Allocation is based on clinical or laboratory test findings* |
|  | ***Unclear*** *– Study uses statements such as “we randomly allocated” or “using a randomised design”, but insufficient detail is given of the process to allow a decision to be made regarding the adequacy of the method.* | |
| 1. **Was the treatment allocation adequately concealed?** | ***YES*** *– participants and physicians could not foresee allocation assignment, because an adequate method was used to conceal allocation. Adequate allocation concealment methods include:*   - *Central allocation* - *Identical drug containers are sequentially numbered* - *Opaque, sealed, sequentially numbered assignment envelopes are used* - *Remotely labelled study kit with no indication of treatment within* - *Statement that allocation was concealed from investigators* | ***NO*** *– participants or physicians could potentially foresee allocation. Inadequate allocation concealment procedures include:*   - *The use of an open random allocation schedule* - *Assignment envelopes are not appropriate, such that envelopes may be unsealed, not sequentially numbered, or see-through* - *Rotation or alternation* - *Methods based on date of birth, case record number, or other patient identifiers* |
|  | ***Unclear*** *– No mention of precautions taken to conceal treatment allocation.* | |
| 1. **Were participants blinded to the intervention?** | ***YES*** *– patients were adequately blinded and it is unlikely that the blinding could be broken. A statement describing that the treatments were identical or “matched” is expected to allow a decision.* | ***NO*** *– No blinding, incomplete blinding, or blinding attempted but likely to have been broken* |
|  | ***Unclear*** *– Study reported as single- or double-blinded but no description of the process of blinding, thereby not allowing a decision to be made regarding the adequacy.* | |
| 1. **Were physicians blinded to the intervention?** | ***YES*** *– physicians were adequately blinded and it is unlikely that the blinding could be broken. If the treatments are identical a statement that the physicians were blinded allows a yes* | ***NO*** *– No blinding, incomplete blinding, or blinding attempted but likely to have been broken* |
|  | ***Unclear*** *– Study reported as single- or double-blinded but no description of the process of blinding, thereby not allowing a decision to be made regarding the adequacy.* | |
| 1. **Were outcome assessors blinded to the intervention?** | ***YES*** *– treatment choice is not evident when measuring outcome.*   - *If outcome is patient-reported, then the answer for “Were participants blinded to the intervention?” will be the same for this question.* - *For physician-assessed outcomes, this is dependent on whether the treatment allocation can be identified from examination of the patient or their tests* | ***NO*** *– treatment choice is likely to be evident when measuring outcome*   - *If outcome is patient-reported, then the answer for “Were participants blinded to the intervention?” will be the same for this question.* - *For physician-assessed outcomes, this is dependent on whether the treatment allocation can be identified from examination of the patient or their tests* |
| 1. **Incomplete outcome data: Is the attrition rate <15%?** | ***YES*** *– drop-out rate is less than 15%* | ***NO*** *– drop-out rate is greater than 15%* |
|  | ***Unclear*** *– flow of patients not given. Unable to ascertain number of withdrawals* | |
| 1. **Are all pre-specified outcomes of interest reported in the pre-specified way?** | ***YES*** *– all the study’s pre-specified outcomes of interest are reported in the pre-specified way* | ***NO*** *– Outcome measures were not pre-specified, such as:*   - *Not all the pre-specified outcomes have been reported* - *Outcomes are not reported using the measurements or methods pre-specified* - *Outcomes reported had not been pre-specified, such as post-hoc analyses* - *Outcomes of interest have been incompletely reported* - *The report does not report outcomes that would be expected from such a study* |
|  |  |  |
| 1. **Was intention-to-treat analysis used?** | ***YES*** *– all randomised participants are analysed according to the group they were allocated to, regardless of non-compliance, protocol violations, drop-outs etc.* | ***NO*** *– the study states that ITT was used, but patients have been excluded from analysis, post-randomisation, for variety of reasons. Alternatively, per-protocol analysis is used to analyse only participants that adhered fully to their allocated treatment, excluding any drop-outs or moves between treatment groups.* |
|  | ***Unclear*** *– does not mention if ITT or PP used and n for each outcome not presented* | |
| 1. **Were the treatment and control group similar at baseline?** | ***YES*** *– treatment and control group were similar regarding patient demographics and baseline parameters.* | ***NO*** *– Treatment and control groups were dissimilar with regards to patient demographics and baseline parameters. Alternatively, characteristics are not presented in either text or table* |
|  | ***Unclear****- if characteristics are mentioned in text, but data are not presented* | |
